# A first-in-class TIMM44 blocker inhibits bladder cancer cell growth

**DOI:** 10.1038/s41419-024-06585-x

**Published:** 2024-03-11

**Authors:** Lifeng Zhang, Xiaokai Shi, Lei Zhang, Yuanyuan Mi, Li Zuo, Shenglin Gao

**Affiliations:** 1 Department of Urology, The Affiliated Changzhou Second People’s Hospital of Nanjing Medical University, Changzhou, China; 2 Department of Urology, Changzhou Second People’s Hospital, Changzhou Medical Center, Nanjing Medical University, Nanjing, China; 3 Department of Urology, Affiliated Hospital of Jiangnan University, Wuxi, China; 4 Department of Urology, Gonghe County Hospital of Traditional Chinese Medicine, Hainan Tibetan Autonomous Prefecture, Qinghai, Province China

**Keywords:** Bladder cancer, Targeted therapies

## Abstract

Mitochondria play a multifaceted role in supporting bladder cancer progression. Translocase of inner mitochondrial membrane 44 (TIMM44) is essential for maintaining function and integrity of mitochondria. We here tested the potential effect of MB-10 (MitoBloCK-10), a first-in-class TIMM44 blocker, against bladder cancer cells. *TIMM44* mRNA and protein expression is significantly elevated in both human bladder cancer tissues and cells. In both patient-derived primary bladder cancer cells and immortalized (T24) cell line, MB-10 exerted potent anti-cancer activity and inhibited cell viability, proliferation and motility. The TIMM44 blocker induced apoptosis and cell cycle arrest in bladder cancer cells, but failed to provoke cytotoxicity in primary bladder epithelial cells. MB-10 disrupted mitochondrial functions in bladder cancer cells, causing mitochondrial depolarization, oxidative stress and ATP reduction. Whereas exogenously-added ATP and the antioxidant N-Acetyl Cysteine mitigated MB-10-induced cytotoxicity of bladder cancer cells. Genetic depletion of TIMM44 through CRISPR-Cas9 method also induced robust anti-bladder cancer cell activity and MB-10 had no effect in TIMM44-depleted cancer cells. Contrarily, ectopic overexpression of TIMM44 using a lentiviral construct augmented proliferation and motility of primary bladder cancer cells. TIMM44 is important for Akt-mammalian target of rapamycin (mTOR) activation. In primary bladder cancer cells, Akt-S6K1 phosphorylation was decreased by MB-10 treatment or TIMM44 depletion, but enhanced after ectopic TIMM44 overexpression. In vivo, intraperitoneal injection of MB-10 impeded bladder cancer xenograft growth in nude mice. Oxidative stress, ATP reduction, Akt-S6K1 inhibition and apoptosis were detected in MB-10-treated xenograft tissues. Moreover, genetic depletion of TIMM44 also arrested bladder cancer xenograft growth in nude mice, leading to oxidative stress, ATP reduction and Akt-S6K1 inhibition in xenograft tissues. Together, targeting overexpressed TIMM44 by MB-10 significantly inhibits bladder cancer cell growth in vitro and in vivo.

## Introduction

Bladder cancer is still one of the most common tumor in the population [1, 2] and the long-term survival rate of patients with advanced disease (i.e., metastatic cancer) is still very low [3, 4]. The main treatment for non-muscle-invasive bladder cancer is complete tumor resection combined with intravesical vaccines, chemotherapy and maintenance immunotherapy [5–7]. Treatment options for muscle-invasive bladder cancer mainly include radical cystectomy and neoadjuvant chemotherapy, along with other treatments [5–7], with immunotherapy emerging as a viable treatment for patients who fail first-line chemotherapy [5–7]. Bladder cancer is genetically heterogeneous and surrounded by a complex tissue microenvironment [6, 8–10]. Understanding the molecular biology of bladder cancer will help develop new molecularly-targeted therapies [6, 8–10].

Dysregulation of mitochondrial functions can impact bladder cancer progression through several mechanisms. Mitochondria play a multifaceted role in supporting the survival, proliferation and metastatic potential of bladder cancer cells, making them a crucial target for potential therapeutic interventions [9, 11–14]. Altered mitochondrial energy metabolism, apoptotic resistance, dysregulated production of reactive oxygen species (ROS) and mitochondrial DNA mutations all contribute to the progression of bladder cancer [9, 11, 12]. It is therefore important to identify key mitochondrial genes that are dysregulated in bladder cancer and to explore possible targeted therapies.

Translocase of inner mitochondrial membrane 44 (TIMM44) locating at the inner mitochondrial membrane (IMM) is a key component of protein associated motor (PAM) complex, which also include TIMM23 [15, 16]. TIMM44 is proposed to anchor mitochondrial heat shock protein 70 (mtHsp70) to TIMM23 PAM complex and this process is ATP-dependent [17–19]. TIMM44 is essential for mitochondrial pre-protein import into the mitochondrial matrix [17, 18]. Yu et al., have shown that antigen R-TIMM44 association is vital for the *TIMM44* mRNA stability and growth of ovarian cancer cells [20]. TIMM44 silencing, however, suppressed ovarian cancer cell growth [20]. Bonora et al., identified two novel variants in *TIMM44*, which are associated with thyroid carcinoma progression [16]. Guo et al., have recently shown that TIMM44 is upregulated in human glioma and is vital for in vitro and intracranial growth of glioma cells [21]. Nevertheless, expression and potential functions of TIMM44 in bladder cancer have not extensity studied.

MB-10 (MitoBloCK-10) is a first-in-class TIMM44 blocker [21–23]. Molecular docking, molecular dynamics modeling and mutational analyses reveal that MB-10 binds to a specific pocket in the C-terminal domain of TIMM44 of the PAM complex, thereby blocking mitochondrial pre-protein import [21–23]. Few studies have researched a possible anti-cancer activity of MB-10. Guo et al., found that TIMM44 blockage by MB-10 inhibited glioma cell viability, proliferation and migration in vitro [21]. Ma et al., recently reported that the TIMM44 blocker induced anti-angiogenic activity in endothelial cells [22]. We here tested its potential effect on bladder cancer cells and explored the possible underlying mechanisms.

## Materials and methods

### Reagents and chemicals

MB-10 was provided by Dr. Cao [21]. Cell Counting Kit-8 (CCK-8) was supplied by Dojindo Co. (Kumamoto, Japan). Puromycin, polybrene, medium, antibiotics, serum, AZD5363, chloroquine (Cq), 3-Methyladenine (3-MA), ATP, N-Acetyl-L-cysteine (NAC), RNA reagents and caspase inhibitors were procured from Sigma-Aldrich Chemicals (St. Louis, MO). Additionally, EdU (5-ethynyl-2’-deoxyuridine), DAPI (4’,6-diamidino-2-phenylindole), TUNEL (terminal deoxynucleotidyl transferase dUTP nick end labeling), MitoSOX, JC-1, and CellROX fluorescence dyes were acquired from Invitrogen Thermo-Fisher (Shanghai, China). All antibodies and mRNA primers were from Dr. Cao [21, 24, 25].

### Human tissues

A total of twelve (12) muscle invasive bladder cancer (T2-T4a, N0, M0) patients (65–76 years old, half male hale female) with tumor resection surgery were enrolled. The resected cancer tissues and surrounding normal bladder tissues were carefully isolated under the microscopy. The tissue lysates were subject to Western blotting and qRT-PCR (reverse transcription quantitative real-time PCR) assays. All patients were administrated at the affiliated Changzhou No.2 People’s Hospital of Nanjing Medical University and written-informed consent was obtained from each patient. The protocols for using human tissues were approved by the Ethic Review Board (ERB) of the affiliated Changzhou No.2 People’s Hospital of Nanjing Medical University, according to the principles of the Declaration of Helsinki.

### Cells

The immortalized T24 bladder cancer cells were purchased from Shanghai Institutes for Biological Sciences (Shanghai, China) and were maintained under 8% fetal bovine serum (FBS)-containing Dulbecco’s Modified Eagle’s Medium (DMEM) plus gentamicin, penicillin/streptomycin, L-glutamine and HEPES (Sigma), in a humidified incubator at 37 °C and 5% CO_2_. For primary cancer cells, the bladder cancer tissues and adjacent normal bladder epithelial tissues were carefully isolated and were rinsed with PBS, diced into small fragments, and finely minced. These tissues were then digested by collagenase-I and DNase solution (Sigma) and were then passed through a 70 µm strainer to obtain a single-cell suspension. Subsequently, individual cells were collected, washed, and resuspended in the specified cell culture medium [26]. The primary bladder cancer cells were derived three different patients, namely “priBlCa-1/priBlCa-2/priBlCa-3”, and the primary epithelial cells were derived from two patients, namely “priBEC-1” and “priBEC-2”. To ensure the cell phenotypes, examinations for mycoplasma and microbial contamination, STR profiling, assessment of population doubling time, and evaluation of cell morphology were conducted every 2–3 months. The protocols for using primary human cells were approved by the Ethic Review Board (ERB) of the affiliated Changzhou No.2 People’s Hospital of Nanjing Medical University, according to the principles of the Declaration of Helsinki.

### Western blotting

In brief, 30 µg aliquots of lysed proteins from cellular and tissue samples were separated by 10–12% SDS-PAGE gel and transferred to a PVDF membrane. Following a blocking step with 10% instant non-fat dry milk, the membrane was incubated with specific antibodies overnight at 4 °C, followed by secondary antibody incubation at room temperature for 1 h. The blotting signaling was thereafter visualized using an enhanced chemiluminescence (ECL) machine, and the intensity of each protein band was quantified via ImageJ software. A mitochondria isolation kit (Sigma) was employed to achieve mitochondrial fraction lysates according to the attached protocols. In brief, cells were first homogenized in a hypotonic buffer to disrupt the plasma membrane, and the homogenate was then centrifuged at low speeds to obtain supernatant enriched in mitochondria and a pellet containing other organelles. Subsequently, the mitochondrial fraction was isolated by subjecting the supernatant to high-speed centrifugation. Figure S1 contains uncropped blotting images.

### qRT-PCR

Cellular and tissue RNA was extracted using TRIzol reagents, followed by reverse transcription into cDNA using a cDNA Reverse Transcription Kit (Takara). Subsequently, qRT-PCR reactions were conducted using SYBR Green PCR Master Mixes (Thermo Fisher) on the ABI-7900 system (Applied Biosystems, Shanghai, China). *GAPDH* (*Glyceraldehyde-3-phosphate dehydrogenase*) was tested as a reference gene for normalization, and the 2^-ΔΔCt^ method was employed to determine the relative gene expression levels. All verified primers were provided by Dr. Cao [21].

### Cell Counting kit (CCK-8) viability and cell death assay

Cells (at 3 × 10^3^ cells/well) were seeded into 96-well plates and were subject to the designated treatments for 72 h. Thereafter, 15 µL of CCK-8 reagent per well was added for 2 h, with CCK8 absorbance tested at 450 nm under a microplate reader (Thermo Fisher Scientific, Shanghai, China). Alternatively, an automatic cell counter (Roche, Shanghai, China) was utilized to quantify cells showing positive Trypan blue staining, indicating cell death.

### Thymidine DNA ELISA

Cells with the designated treatments were cultured in the presence of [H^3^]-labeled thymidine, which was incorporated into newly synthesized DNA. Cells were then harvested and lysed to release DNA. An ELISA kit (from Dr. Song [27]) was thereafter utilized to detect [H^3^]-labeled DNA, and the absorbance was detected.

### Transwell assay

Transwell plates with 8 μm of pore size (BD Biosciences, Shanghai, China) were utilized in the study. Briefly, in the upper chamber, 2 × 10^4^ cells/well were seeded in 350 µL of medium containing 0.5% FBS. The lower chamber was filled with 600 µL of media containing 8% FBS. After 24 h, the cells in the upper chamber were removed with a cotton swab. The cells on the lower surface were fixed (by 70% ethanol), washed (with PBS), stained (with 0.5% crystal violet), photographed and counted. For in vitro cell invasion assays, Transwell inserts were always coated with 0.5 mg/mL of Matrigel (BD Biosciences).

### Nuclear fluorescence staining

Cells (at 2 × 10^4^ cells/well) were seeded into 12-well plates and were subject to the designated treatments for 48 h. Cells were then stained with TUNEL (terminal deoxynucleotidyl transferase dUTP nick end labeling) or EdU (5-ethynyl-2′-deoxyuridine) dye. Afterwards, cells were harvested, fixed (by 70% ethanol) and permeabilized (by 0.5% Triton-X-100). DAPI was then added for counterstaining. The fluorescence signaling was detected under a confocal microscopy (Leica, Wetzlar, Germany).

### Cellular fluorescence staining assays

Cells (at 2 × 10^4^ cells/well) were seeded into 12-well plates and were subject to the designated treatments for 24 h. Cells were further stained with the cationic carbocyanine fluorescence dye JC-1, the mitochondrial superoxide dye MitoSOX green or the CellROX deep red. Afterwards, cells were harvested, fixed (by 70% ethanol) and permeabilized (by 0.5% Triton-X-100). The fluorescence signaling was detected under a confocal microscopy (Leica). The fluorescence intensities were always quantified by ImageJ software.

### Caspase activity

In brief, total cellular lysates or tissue lysates were measured by Caspase fluorescent assay kits (BD bioscience, Suzhou, China). Caspase-3 and Caspase-9 activities were tested fluorometrically with the corresponding active substrates, Ac-DEVD-AMC (7-amido-4-methyl coumarin) and Ac-LEHD-AMC (7-amido-4-methyl coumarin).

### Flow cytometry

In brief, the bladder cancer cells or epithelial cells were first centrifuged, resuspended and subsequently stained with Annexin V-APC (5 µL) and/or propidium iodide (PI, 5 µL) (Sigma). The CytoFLEX flow cytometry machine (Beckman, Shanghai, China) was then employed to assess cell apoptosis or cell cycle progression.

### GSH/GSSG ratio

The ratio of reduced glutathione (GSH) to oxidized glutathione (GSSG), reflecting cellular redox status, was measured by a commercial kit (Sigma) based on the attached protocols. GSH is derivatized to 5,5’-dithiobis(2-nitrobenzoic acid) (DTNB), producing a yellow compound that was quantified spectrophotometrically at 450 nm. 30 μL of cellular or tissue lysates per treatment were tested.

### The mitochondrial complex I activity and ATP assays

The enzymatic activity of complex I in mitochondrial fraction lysates was measured by a commercial kit (Sigma), which employs spectrophotometry to monitor the conversion of NADH to NAD^+^ by complex I. The resultant decline in absorbance at 350 nm was recorded as a direct indicator of complex I activity. The cellular and tissue ATP contents were measured by a commercial colorimetric kit (Sigma) based on the attached protocol. Thirty μL of cellular or tissue lysates per treatment were tested.

### CRISPR/Cas9-mediated TIMM44 knockout (KO)

Cells were first infected with lentivirus encoding the CRISPR-associated endonuclease 9 (Cas9)-expressing construct [21]. Stable cells were formed after selection and were further transduced with the lentiviral CRISPR/Cas9-TIMM44-KO construct (with puromycin selection gene but not GFP-Tag [21]). Stable KO-TIMM44 cells were then formed after puromycin selection and cells were distributed into 96-well plates. After TIMM44 KO verification, single stable KO-TIMM44 cells were established. The control Cas9-expressing cells were transduced with the lentiviral CRISPR/Cas9-control construct (“KO-C”).

### TIMM44 overexpression

Cells were first infected with the lentivirus encoding the TIMM44-overexpressing construct (“oe-TIMM44” from Dr. Cao [21]). The construct contains puromycin selection gene but not GFP-Tag. Afterwards, puromycin-containing medium was added for another 5–6 passages. Thereafter, two stable cell selections, namely “oe-TIMM44-stb slc1” and “oe-TIMM44-stb slc2”, were formed. Control cells were stably transduced with the lentiviral empty vector (“Vec”).

### Akt1 mutation

Dr. Chen [28] generously supplied lentiviral particles harboring the constitutively-active S473D mutant Akt1 (caAkt1), which were introduced into cultured bladder cancer cells. The stable cells expressing caAkt1 were established by employing puromycin-based selection.

### Xenograft studies

The athymic nude mice, comprising an equal distribution of both male and female individuals aged 5–6 weeks and weighing between 17.8 and 18.2 g, were sourced from the Shanghai Laboratory Animal Center (SLAC, Shanghai, China). These mice were subcutaneously (*s.c*.) injected with six million priBlCa-1 primary bladder cancer cells suspended in 100 µL of Matrigel basic medium, leading to the establishment of xenograft tumors with volumes approaching 100 mm³ within three weeks. Subsequently, the mice were divided into two groups, one receiving intraperitoneal injection of MB-10 at a dose of 20 mg/kg body weight every 48 h for a total of three doses, while the other group received vehicle control (PBS + Tween 80). The mice body weights and the tumor volumes [*π*/6 × larger diameter × (smaller diameter)^2^] were recorded every six days. Estimated daily tumor growth (in mm^3^ per day) was also calculated as described [29]. Alternatively, TIMM44-KO or KO-C priBlCa-1 primary bladder cancer cells (six million cells suspended in 100 µL of Matrigel basic medium) were *s.c*. injected to the flanks of the nude mice. Xenografts were formed 50 days after cell injection and were tested thereafter. The animal studies were approved by Institutional Animal Care and Use Committee and Ethics Committee of the affiliated Changzhou No.2 People’s Hospital of Nanjing Medical University.

### Tissue fluorescence assay

In brief, the procedures involved heating paraffin-embedded tissue sections at 60 °C for 2 h. Following dewaxing and hydration, the tissue slices were immersed in citric acid buffer at 95 °C for 15 min and subsequently rinsed three times with PBS. To prevent non-specific binding, goat serum was applied to block the tissue slices for 20 min at 37 °C. Next, the tissue slices were incubated with the specified TUNEL and DAPI fluorescence dyes, washed, and examined using a confocal microscope (ZEISS).

### Statistical analysis

Throughout all in vitro experiments, the investigators conducted blinded assessments regarding group allocations. The in vitro experiments were replicated five times. Data exhibiting a normal distribution were expressed as the mean ± standard deviation (SD). Statistical analysis employed SPSS version 23.0 (SPSS Co., Chicago, IL). To compare two specific groups, an unpaired Student’s *t* test was utilized. For comparisons involving more than two groups, the one-way ANOVA with the Scheffe’ and Tukey Test was applied. Statistical significance was attributed to *P* < 0.05.

## Results

### TIMM44 overexpression in human bladder cancer tissues and cells

**Fig. 1:**
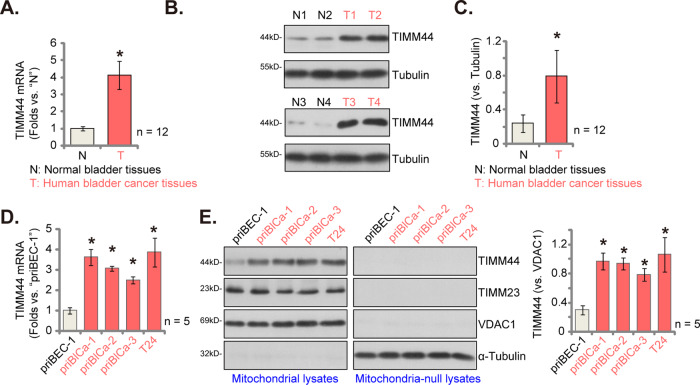
TIMM44 is overexpression in human bladder cancer tissues and cells. *TIMM44* mRNA (**A**) and protein (**B**, **C**) expression in the described human bladder cancer tissues (“T”) and surrounding normal bladder tissue (“N”) tissues was tested. *TIMM44* mRNA expression in both primary (priBlCa-1/priBlCa-2/priBlCa-3) and immortalized (T24) bladder cancer cells as well as in priBEC-1 bladder epithelial cells was shown (**D**). The mitochondrial fraction lysates and mitochondria-null lysates of the above cells were obtained and expression of listed protein was tested (**E**). The data were presented as mean ± standard deviation (SD). In (**A**–**C**), 12 sets of patients tissues were in each group (*n* = 12). For (**D**, **E**) *n* = 5 stands for five biological repeats. * *P* < 0.05 vs. “N” tissues/priBEC-1 cells.

We first tested TIMM44 expression in human bladder cancer tissues. A total of twelve (12) bladder cancer patients with tumor resection surgery were enrolled. The cancer tissues (“T”) and surrounding normal bladder tissue (“N”) tissues were carefully isolated. Part of the tissues were homogenized through tissue lysis buffer. As shown, *TIMM44* mRNA expression in “T” cancer tissues were over four folds of that in the “N” normal tissues (Fig. 1A). Moreover, TIMM44 protein upregulation was detected in bladder cancer tissues of four representative patients (“T1/T2/T3/T4”, Fig. 1B), and its expression was relatively low in corresponding normal tissues (“N1/N2/N3/N4”, Fig. 1B). When combining the blotting results from all twelve groups of tissues, we show that TIMM44 protein upregulation is significant in the bladder cancer tissues (Fig. 1C).

Whether TIMM44 is overexpressed in human bladder cancer cells was also examined. As shown, *TIMM44* mRNA expression is substantially elevated in both primary (priBlCa-1/priBlCa-2/priBlCa-3) and T24 immortalized cancer cells (Fig. 1D), with relative low mRNA expression detected in priBEC-1 bladder epithelial cells (Fig. 1D). Moreover, TIMM44 protein upregulation was observed in the mitochondrial fraction lysates of the primary and immortalized cancer cells (Fig. 1E), and low expression in priBEC-1 epithelial cells (Fig. 1E). The mitochondrial fraction lysates contain voltage dependent anion channel 1 (VDAC-1) but no α-Tubulin (Fig. 1E). Notably, TIMM44 protein is not present in the mitochondria-null lysates (no VDAC1 but with α-Tubulin, Fig. 1E). TIMM23 is another fundamental component in the mitochondrial protein import machinery, associated to TIMM44 [17, 18]. As shown, TIMM23 protein expression is not significantly different between epithelial cells and primary/immortalized bladder cancer cells (Fig. 1E). Thus, TIMM44 is overexpression in both human bladder cancer tissues and cells.

### MB-10 exerts significant anti-bladder cancer cell activity, inhibiting cell viability, proliferation and mobility

**Fig. 2:**
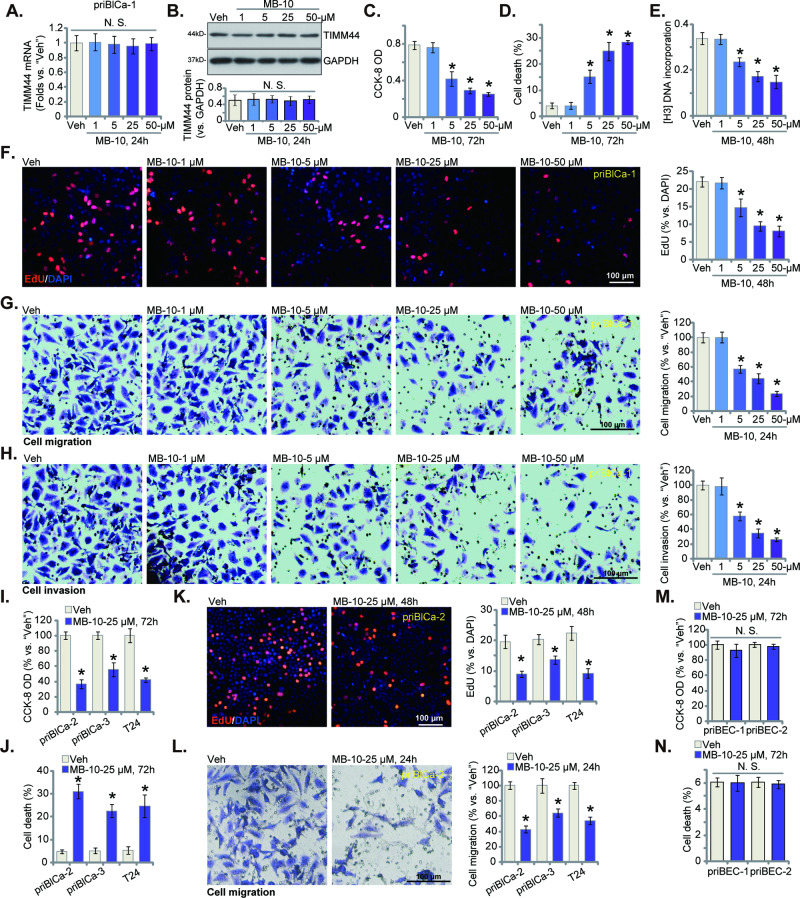
MB-10 exerts significant anti-bladder cancer cell activity, inhibiting cell viability, proliferation and mobility. The priBlCa-1 primary bladder cancer cells were maintained under complete medium, treated with MB-10 at designated concentration and cultivated for indicated hours, *TIMM44* mRNA and protein expression was tested (**A**, **B**). Cell viability and death were tested by CCK-8 (**C**) and Trypan blue staining (**D**) assays, respectively. Cell proliferation was measured via [H^3^] DNA incorporation assay (**E**) and nuclear EdU staining assay (**F**), with in vitro cell migration (**G**) and invasion (**H**) tested via “Transwell” assays. The primary bladder cancer cells, priBlCa-2 and priBlCa-3 (derived from two other patients) (**I**–**L**), T24 immortalized cells (**I**–**L**) or the primary human bladder epithelial cells (priBEC-1 and priBEC-2) (**M**, **N**) were maintained under complete medium, treated with MB-10 (25 μM) and cultivated for indicated hours, cell viability (CCK-8 assay, **I**, **M**), death (Trypan blue staining assay, **J**, **N**), proliferation (nuclear EdU staining assay, **K**) and migration (“Transwell” assay, **L**) were tested. “Veh” stands for the vehicle control treatment (0.1% DMSO). The data were presented as mean ± standard deviation (SD). *n* = 5 stands for five biological repeats. * *P* < 0.05 vs. “Veh”. “N. S.” stands for non-statistical difference (*P* > 0.05). The in vitro experiments were repeated five times with similar results obtained. Scale bar = 100 μm.

First, the primary human bladder cancer cells, priBlCa-1, were treated with MB-10 at increasing concentrations (from 1 to 50 μM). *TIMM44* mRNA (Fig. 2A) and protein (Fig. 2B) expression was unchanged by the applied MB-10 treatment. Yet, the TIMM44 blocker dose-dependently inhibited priBlCa-1 cell viability and decreased CCK-8 optical density (OD) (Fig. 2C). Evidenced by increased Trypan blue staining results, we showed that MB-10 dose-dependently induced priBlCa-1 cell death (Fig. 2D). The [H^3^] DNA incorporation (Fig. 2E) and nuclear EdU incorporation (Fig. 2F) were both significantly decreased following 5–50 μM of MB-10 treatment, supporting the anti-proliferative activity by the TIMM44 blocker. The mobility of priBlCa-1 cells was also tested via “Transwell” studies. MB-10 dose-dependently inhibited in vitro migration (Fig. 2G) and invasion (Fig. 2H) of priBlCa-1 cells. The titration experiments showed that 25 μM of MB-10, among the tested concentrations, exerted significant anti-bladder cancer cell activity, and inhibiting cell viability, proliferation and mobility (Fig. 2C–H). This concentration was therefore chosen for the following experiments.

Next we examined the effect of MB-10 in other bladder cancer cells, including the primary cancer cells from two other patients, priBlCa-2 and priBlCa-3, as well as in the immortalized T24 cells. The TIMM44 blocker, at 25 μM, decreased cell viability in the primary and immortalized bladder cancer cells (Fig. 2I). Significant cancer cell death was induced after MB-10 treatment, supported by the increased Trypan blue staining (Fig. 2J). Cell proliferation, tested by nuclear EdU incorporation (Fig. 2K), and in vitro migration (Fig. 2L) were also substantially inhibited after treatment of TIMM44 blocker in the bladder cancer cells. The potential effect of MB-10 on non-cancerous epithelial cells was explored as well. In the primary human bladder epithelial cells, priBEC-1 and priBEC-2, 25 μM of MB-10 failed to induce significant viability reduction (Fig. 2M) and cell death (Fig. 2N). These results confirm its selective effect only in cancerous cells.

### MB-10 induces mitochondrial apoptosis cascade activation in bladder cancer cells

**Fig. 3:**
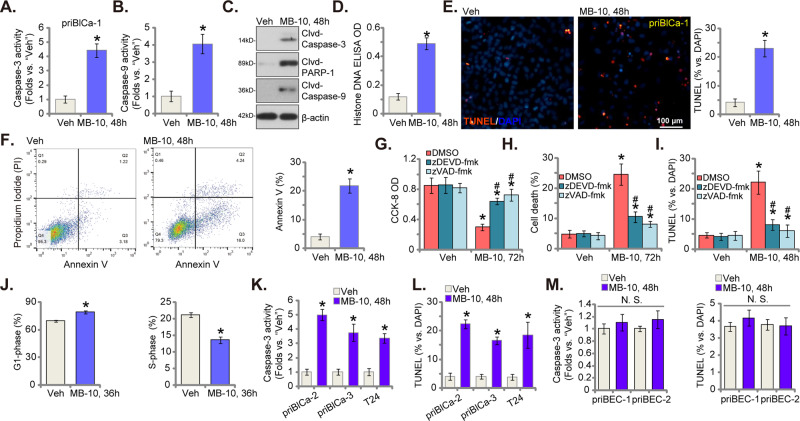
MB-10 induces mitochondrial apoptosis cascade activation in bladder cancer cells. The priBlCa-1 primary bladder cancer cells were maintained under complete medium, treated with MB-10 (25 μM) and cultivated for indicated hours, Caspase-3 activity (**A**), Caspase-9 activity (**B**) and expression of apoptosis-related proteins (**C**) were tested. Histone-bound DNA contents were measured by ELISA (**D**); Cell apoptosis was measured via TUNEL-nuclei fluorescence staining (**E**) and Annexin V-propidium iodide (PI) flow cytometry (**F**) assays; Cell cycle progression was measured via PI flow cytometry assays (**J**); The priBlCa-1 primary bladder cancer cells were pretreated for 45 min with the Caspase-3 specific inhibitor zDEVD-fmk (50 μM) or the pan Caspase inhibitor zVAD-fmk (50 μM), followed by MB-10 (25 μM) stimulation, cells were further cultivated for indicated time periods, cell viability, death and apoptosis were tested by CCK-8 (**G**), Trypan blue staining (**H**) and TUNEL-nuclei fluorescence staining (**I**) assays, respectively. The primary bladder cancer cells (priBlCa-2 and priBlCa-3), T24 immortalized cells or the primary human bladder epithelial cells (priBEC-1 and priBEC-2) were maintained under complete medium, treated with MB-10 (25 μM) and cultivated for indicated hours, Caspase-3 activity (**K**, **M**) and apoptosis (TUNEL-nuclei fluorescence staining, **L**, **M**) were tested. “Veh” stands for the vehicle control treatment (0.1% DMSO). The data were presented as mean ± standard deviation (SD). *n* = 5 stands for five biological repeats. *P < 0.05 vs. “Veh”. “N. S.” stands for non-statistical difference (*P* > 0.05). ^#^*P* < 0.05 vs. “DMSO” pre-treatment (**G**–**I**). The in vitro experiments were repeated five times with similar results obtained. Scale bar = 100 μm.

MB-10 is a TIMM44 blocker, we show that the mitochondrial apoptosis cascade is induced by MB-10 in bladder cancer cells. Both Caspase-3 activity (Fig. 3A) and Caspase-9 activity (Fig. 3B) were significantly increased in MB-10 (25 μM)-treated priBlCa-1 primary cancer cells. The TIMM44 blocker also provoked cleavages of Caspase-3, Caspase-9 and Poly (ADP-ribose) polymerase (PARP-1) in priBlCa-1 cells (Fig. 3C). Moreover, increased Histone-bound DNA supported increased DNA breaks in MB-10-treated priBlCa-1 cells (Fig. 3D). MB-10 induced apoptosis activation in priBlCa-1 cells and the TUNEL-positively stained nuclei percentage was significantly increased (Fig. 3E). Furthermore, flow cytometry assay results showed that Annexin V positively-stained priBlCa-1 cells were substantially increased after MB-10 treatment (Fig. 3F). Importantly, apoptosis inhibitors mitigated MB-10-induced cytotoxicity in priBlCa-1 cells. The Caspase-3 specific inhibitor zDEVD-fmk or the pan Caspase inhibitor zVAD-fmk largely ameliorated MB-10 (25 μM)-induced viability (CCK-8 OD) reduction (Fig. 3G), cell death (Trypan blue assays, Fig. 3H) and apoptosis (TUNEL assays, Fig. 3I). Therefore, the mitochondrial apoptosis cascade induction should be a primary mechanism of MB-10-induced cytotoxicity in bladder cancer cells.

The propidium iodide (PI) flow cytometry results, Fig. 3J, further showed that MB-10 (25 μM) induced G1-S arrest in priBlCa-1 primary bladder cancer cells, by increasing G1-phase cell percentage and decreasing S-phase cell percentage (Fig. 3J). MB-10 treatment also activated autophagy in priBlCa-1 cells, which was supported by Light Chain 3B-I LC3B-I to LC3B-II transition, p62 protein degradation, and Beclin-1 upregulation (Figure S2A). However, two autophagy inhibitors, chloroquine (Cq) and 3-methyladenine (3-MA), failed to significantly affect MB10-induced viability reduction (Figure S2B) and death (Fig. 2C) in priBlCa-1 cells. The TIMM44 blocker also induced apoptosis in other bladder cancer cells. In priBlCa-2 and priBlCa-3 primary cancer cells and immortalized T24 cells, MB-10 (25 μM) treatment enhanced the Caspase-3 activity (Fig. 3K) and increased the number of nuclei with positive TUNEL staining (Fig. 3L). Contrarily, in the primary human bladder epithelial cells, priBEC-1 and priBEC-2, treatment with the TIMM44 blocker failed to activate Caspase-3 and provoke apoptosis (Fig. 3M).

### MB-10 impairs mitochondrial functions in bladder cancer cells

**Fig. 4:**
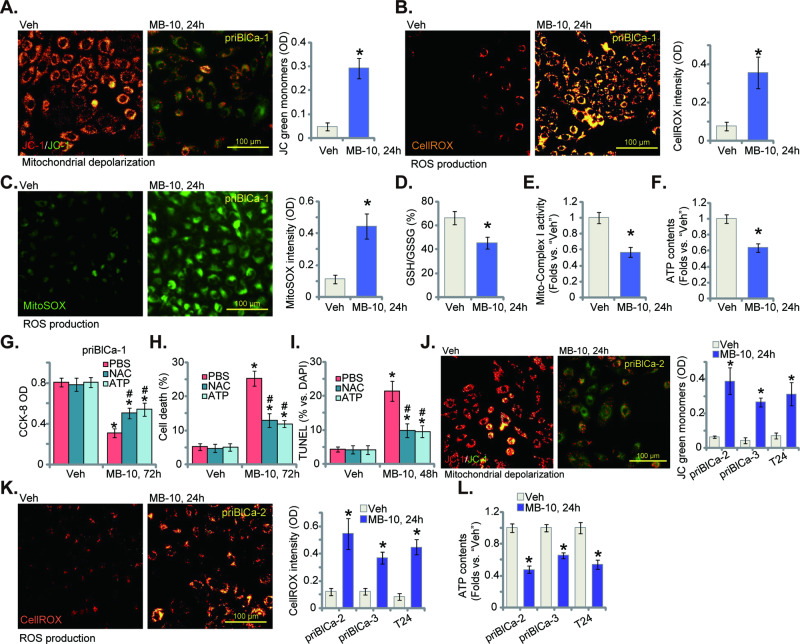
MB-10 impairs mitochondrial functions in bladder cancer cells. The priBlCa-1 primary bladder cancer cells were maintained under complete medium, treated with MB-10 (25 μM) and cultivated for indicated hours, mitochondrial depolarization was measured via JC-1 staining assay (**A**); Mitochondrial ROS production was measured by CellROX fluorescence staining (**B**) and MitoSOX fluorescence staining (**C**) assays; GSH/GSSG ratio was also measured (**D**); The mitochondrial complex I activity (**E**) and cellular ATP contents (**F**) were tested as well. The priBlCa-1 cells were pretreated for 45 min with ATP (1 mM) or NAC (400 μM), followed by MB-10 (25 μM) stimulation, cells were further cultivated for indicated time periods, cell viability, death and apoptosis were tested by CCK-8 (**G**), Trypan blue staining (**H**) and TUNEL-nuclei fluorescence staining (**I**) assays, respectively. The primary bladder cancer cells (priBlCa-2 and priBlCa-3) or T24 immortalized cells were maintained under complete medium, treated with MB-10 (25 μM) and cultivated for indicated hours, mitochondrial depolarization was measured via JC-1 staining assay (**J**); ROS production was measured by CellROX staining assays (**K**), with cellular ATP contents tested as well (**L**). “Veh” stands for the vehicle control treatment (0.1% DMSO). The data were presented as mean ± standard deviation (SD). *n* = 5 stands for five biological repeats. *P < 0.05 vs. “Veh”. ^#^*P* < 0.05 vs. PBS pretreatment (**G**–**I**). The in vitro experiments were repeated five times with similar results obtained. Scale bar = 100 μm.

TIMM44 is vital for maintaining mitochondrial integrity and functions, we next explored mitochondrial function change in bladder cancer cells. In priBlCa-1 primary cancer cells, MB-10 (25 μM) induced mitochondrial depolarization, evidenced by the conversion of JC-1 red fluorescence aggregates to green fluorescent monomers (Fig. 4A). The increases in CellROX fluorescence intensity (Fig. 4B) and MitoSOX Green fluorescence intensity (Fig. 4C) supported mitochondrial ROS production and oxidative stress in MB-10-stimulated priBlCa-1 cells. Moreover, the ratio of reduced GSH to oxidized GSH (GSSG) (GSH/GSSG%) was significantly decreased in priBlCa-1 cells after treatment with the TIMM44 blocker (Fig. 4D). Importantly, MB-10 decreased mitochondrial complex I activity (Fig. 4E) and reduced cellular ATP contents (Fig. 4F). These results together supported that MB-10 disrupts mitochondrial functions in priBlCa-1 cells.

N-acetyl cysteine (NAC) is widely used as a fast acting antioxidant and cytoprotectant, lowing endogenous oxidant stress and protecting cells against various oxidative insults [30]. We showed that NAC or exogenously-added ATP mitigated MB-10-induced viability reduction, cell death and apoptosis, which were tested by CCK-8 (Fig. 4G), Trypan blue staining (Fig. 4H) and nuclear TUNEL staining (Fig. 4I) assays, respectively. These results supported that mitochondrial function impairment and subsequent oxidative stress are key for MB-10-induced cytotoxicity in bladder cancer cells. MB-10 (25 μM) treatment in priBlCa-2, priBlCa-3 and immortalized T24 cells also provoked mitochondrial depolarization, evidenced again by accumulation of JC-1 green monomers (Fig. 4J). Increased CellROX intensity, supporting ROS production and oxidative stress, was also detected in MB-10-treated primary/immortalized bladder cancer cells (Fig. 4K). ATP contents, on the other hand, was decreased (Fig. 4L).

### TIMM44 knockout exerts significant anti-bladder cancer cell activity

**Fig. 5:**
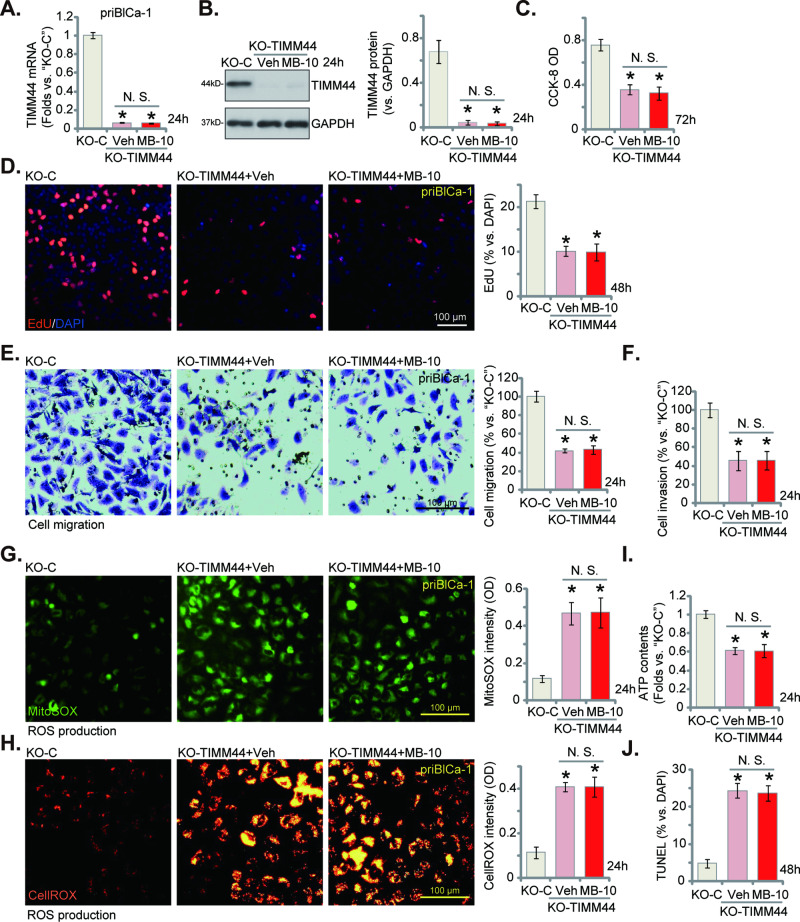
TIMM44 knockout exerts significant anti-bladder cancer cell activity. The priBlCa-1 primary bladder cancer cells with the CRISPR/Cas9-TIMM44-KO construct (“KO-TIMM44”) were treated with or without MB-10 (25 μM) for designated hours; Control cells with the CRISPR/Cas9-KO control construct (“KO-C”) were left untreated. *TIMM44* mRNA and protein expression was tested (**A**, **B**); Cell viability was tested by CCK-8 assay (**C**); Cell proliferation was measured via nuclear EdU staining assay (**D**), and in vitro cell migration (**E**) and invasion (**F**) tested via “Transwell” assays; The mitochondrial ROS production was measured by CellROX/MitoSOX fluorescence staining assays (**G**, **H**), with ATP contents measured as well (**I**). Cell apoptosis was tested by TUNEL-nuclei staining assays (**J**). The data were presented as mean ± standard deviation (SD). *n* = 5 stands for five biological repeats. *P < 0.05 vs. “KO-C”. “N. S.” stands for non-statistical difference (*P* > 0.05). The in vitro experiments were repeated five times with similar results obtained. Scale bar = 100 μm.

Since blockade of TIMM44 induces remarkable antitumor activity in bladder cancer cells, we propose that genetic depletion of TIMM44 should mimic MB-10’s activity. Therefore, CRISPR/Cas9 method was employed to knockout TIMM44. Specifically, a lentiviral CRISPR/Cas9-TIMM44-KO construct (from Dr. Cao’s group [21]) was transduced to Cas9-expressing priBlCa-1 primary cancer cells. The single stable TIMM44 KO cells, namely “KO-TIMM44”, were then formed after puromycin selection and TIMM44 KO verification. Both mRNA (Fig. 5A) and protein (Fig. 5B) expression of TIMM44 was substantially decreased in TIMM44 KO cells. Using the previously-descried experimental methods, we show that CRISPR/Cas9-induced TIMM44 KO potently inhibited cell viability (CCK-8 OD, Fig. 5C), proliferation (EdU incorporation, Fig. 5D), in vitro migration (Fig. 5E) and invasion (Fig. 5F) in priBlCa-1 cells. Importantly, in KO-TIMM44 cells, MB-10 failed to alter TIMM44 expression (Fig. 5A, B). Nor did it exert further anti-cancer cell activity (Fig. 5C–F). Thus, MB-10 is ineffective in KO-TIMM44 bladder cancer cells.

In priBlCa-1 primary cancer cells, TIMM44 KO also induced mitochondrial dysfunction and caused mitochondrial oxidative stress. Both the MitoSOX green fluorescence intensity (Fig. 5G) and the CellROX red fluorescence intensity (Fig. 5H) were significantly increased in KO-TIMM44 priBlCa-1 cells. ATP contents was reduced in KO-TIMM44 cells (Fig. 5I). TIMM44 KO by the CRISPR/Cas9 method also induced apoptosis activation in priBlCa-1 cells and increased TUNEL-positive nuclei ratio (Fig. 5J). Importantly, MB-10 failed to induce further mitochondrial oxidative stress (Fig. 5G, H), ATP depletion (Fig. 5I) and apoptosis (Fig. 5J) in KO-TIMM44 cancer cells.

### TIMM44 overexpression accelerates proliferation and mobility in bladder cancer cells

**Fig. 6:**
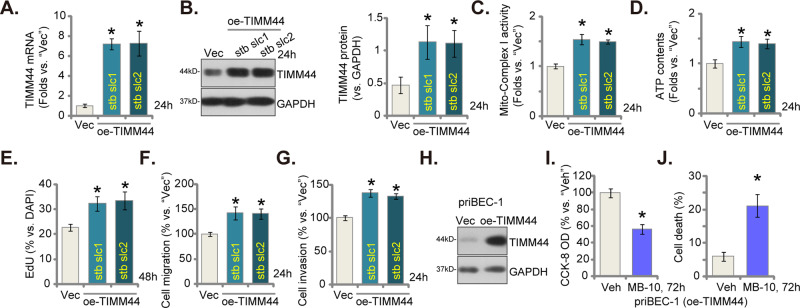
TIMM44 overexpression accelerates proliferation and mobility in bladder cancer cells. The priBlCa-1 primary bladder cancer cells with the lentiviral TIMM44-overexpressing construct (“oe-TIMM44-stb slc1” and “oe-TIMM44-stb slc2”, representing two stable cell selections) or the empty vector (“Vec”) were cultivated for designated time, *TIMM44* mRNA and protein expression was tested (**A**, **B**); The mitochondrial complex I activity (**C**) and cellular ATP contents (**D**) were measured. Cell proliferation (EdU staining assays, **E**), in vitro cell migration (**F**) and invasion (**G**) were tested as well. TIMM44 protein expression in priBEC-1 bladder epithelial cells with the lentiviral TIMM44-overexpressing construct (“oe-TIMM44”) or the empty vector (“Vec”) was shown (**H**). The oe-TIMM44 priBEC-1 cells were treated with MB-10 (25 μM) or vehicle control (“Veh”) for indicated hours, cell viability and death were tested by CCK-8 (**I**) and Trypan blue staining (**J**) assays, respectively. The data were presented as mean ± standard deviation (SD). *n* = 5 stands for five biological repeats. *P < 0.05 vs. “Vec”/ “Veh”. The in vitro experiments were repeated five times with similar results obtained.

Since blockage or depletion of TIMM44 led to robust anti-bladder cancer function, we next propose that increasing TIMM44 expression might produce pro-cancerous activity. To this aim, the lentivirus-packed TIMM44-overexpressing construct (“oe-TIMM44” [21]) were added to priBlCa-1 primary bladder cancer cells. Following selection by puromycin, two stable cell selections, namely “oe-TIMM44-stb slc1” and “oe-TIMM44-stb slc2”, were formed. As compared to control cells with lentiviral empty vector (“Vec”), *TIMM44* mRNA (Fig. 6A) and protein (Fig. 6B) levels were substantially increased in oe-TIMM44-expressing priBlCa-1 cells. Consequently, the mitochondrial complex I activity (Fig. 6C) and cellular ATP contents (Fig. 6D) were both increased. With TIMM44 overexpression, priBlCa-1 cell proliferation, or nuclear EdU-positive nuclei ratio, was augmented (Fig. 6E). The quantified “Transwell” assay results showed that in vitro cell migration (Fig. 6F) and invasion (Fig. 6G) were both accelerated in oe-TIMM44-expressing priBlCa-1 cells.

The TIMM44-overexpressing construct was introduced into non-cancerous epithelial cells (priBEC-1), resulting in the overexpression of TIMM44 (“oe-TIMM44”) (Fig. 6H). This overexpression of TIMM44 enabled MB-10 to induce significant reduction in cell viability (CCK-8 OD) (Fig. 6I) and triggered cell death (Fig. 6J) in the bladder epithelial cells.

### MB-10 inhibits Akt-mTOR activation in bladder cancer cells

**Fig. 7:**
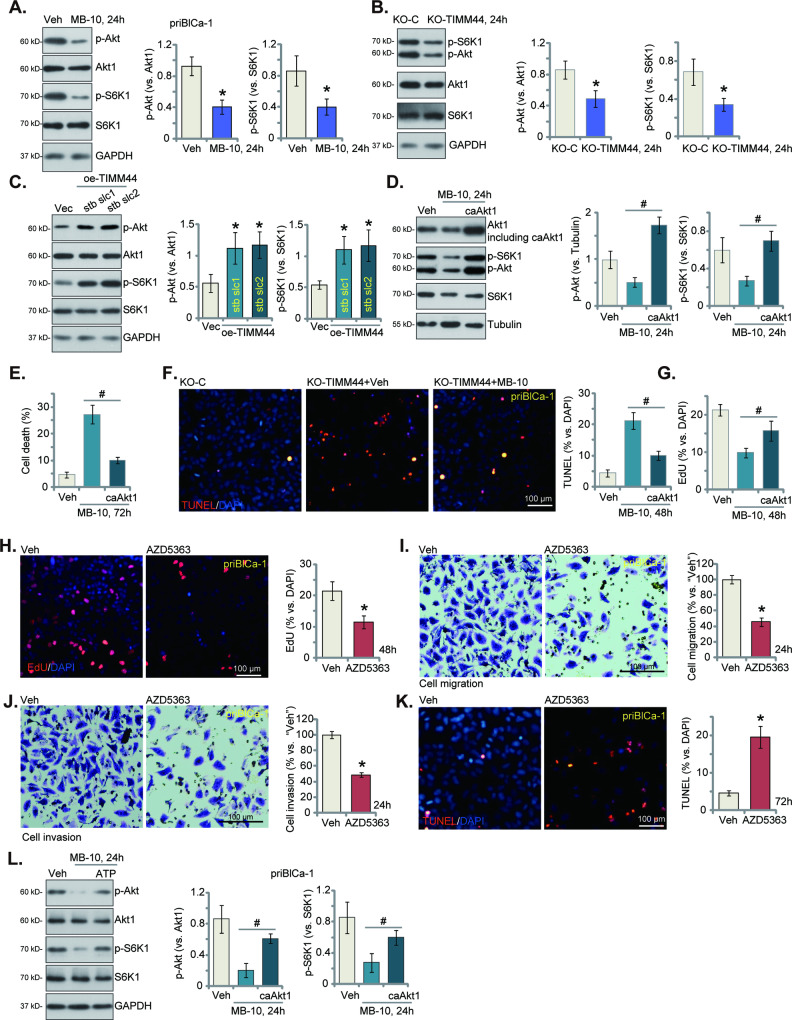
MB-10 inhibits Akt-mTOR activation in bladder cancer cells. The priBlCa-1 primary bladder cancer cells were maintained under complete medium, treated with MB-10 (25 μM) and cultivated for twenty-four hours, expression of listed proteins was tested (**A**). The priBlCa-1 primary bladder cancer cells with the CRISPR/Cas9-TIMM44-KO construct (“KO-TIMM44”), the CRISPR/Cas9-KO control construct (“KO-C”), the lentiviral TIMM44-overexpressing construct (“oe-TIMM44-stb slc1” and “oe-TIMM44-stb slc2”, representing two stable cell selections) or the empty vector (“Vec”), were established and maintained under complete medium for twenty-four hours, expression of listed proteins was shown (**B**, **C**). The priBlCa-1 primary cells with constitutively-active (S473D) mutant Akt1 (caAkt1) were treated with MB-10 for indicated time periods, expression of listed proteins was shown (**D**); Cell death, apoptosis and proliferation were measured via Trypan blue staining (**E**), TUNEL-nuclei staining (**F**) and EdU-nuclei staining (**G**) assays, respectively. The priBlCa-1 primary bladder cancer cells were maintained under complete medium, treated with AZD5363 (2.5 μM) and cultivated for indicated hours. Cell proliferation was measured via nuclear EdU staining assay (**H**), with in vitro cell migration (**I**) and invasion (**J**) tested via “Transwell” assays. Cell apoptosis was tested via TUNEL staining assays (**K**). The priBlCa-1 cells were pretreated for 45 min with ATP (1 mM), followed by MB-10 (25 μM) stimulation, cells were further cultivated for 24 h, and expression of listed proteins was shown (**L**). “Veh” stands for the vehicle control treatment (0.1% DMSO). The data were presented as mean ± standard deviation (SD). *n* = 5 stands for five biological repeats. *P < 0.05 vs. “Veh”/“KO-C”/“Vec” cells. ^#^*P* < 0.05 (**D**–**G**, **L**). The in vitro experiments were repeated five times with similar results obtained. Scale bar = 100 μm.

Activation of Akt-mTOR cascade is vital for the growth of bladder cancer and it is an established therapeutic target [31, 32]. Considering that MB-10 induced significant anti-bladder cancer cell activity, we tested its potential effect on Akt-mTOR cascade activation. Treatment with MB-10 (25 μM) for 24 h significantly decreased phosphorylation of Akt and p70S6 kinase 1 (S6K1) in priBlCa-1 primary bladder cancer cells (Fig. 7A). Total Akt1 and S6K1 protein expression was however unchanged (Fig. 7A). Moreover, CRISPR/Cas9-induced TIMM44 KO (see Fig. 4) also led to Akt-mTOR cascade inactivation in priBlCa-1 cells, decreasing Akt-S6K1 phosphorylation (Fig. 7B). Total Akt-S6K1 protein expression was again unaffected (Fig. 7B). Contrarily, in TIMM44-overexpresed priBlCa-1 cells, “oe-TIMM44-stb slc1” and “oe-TIMM44-stb slc2” (see Fig. 6), Akt-S6K1 phosphorylation was upregulated (Fig. 7C). These results supported that TIMM44 is indeed important for Akt-mTOR activation in the primary bladder cancer cells.

Next, the lentivirus-packed constitutively-active (S473D) mutant Akt1 (caAkt1) was added to priBlCa-1 primary bladder cancer cells, and stable cells established via puromycin-mediated selection. The blotting data in Fig. 7D confirmed expression of caAkt1 (no Taq), that restored Akt-S6K1 phosphorylation in MB-10-treated priBlCa-1 cells (Fig. 7D). Importantly, MB-10-induced priBlCa-1 cell death (Trypan blue staining increase, Fig. 7E), apoptosis (TUNEL-nuclei increasing, Fig. 7F) and proliferation inhibition (EdU-nuclei ratio reduction, Fig. 7G) were largely ameliorated by caAkt1. AZD5363, an orally active and potent pan-AKT kinase inhibitor [33], largely inhibited proliferation (EdU incorporation, Fig. 7H), migration (Fig. 7I) and invasion (Fig. 7J), and activated apoptosis (TUNEL assays, Fig. 7K) in priBlCa-1 cells. These results supported that inactivation of Akt-mTOR cascade is important for MB-10-induced cytotoxicity in primary bladder cancer cells.

ATP is integral to Akt activation because it serves as both the energy source for phosphorylation reactions and the phosphate donor during kinase-mediated phosphorylation events [34–38]. Here we found that exogenously-added ATP mitigated MB-10-induced Akt-mTOR inactivation in priBlCa-1 cells (Fig. 7L). This suggests that reducing ATP levels might be the primary mechanism behind the inhibition of Akt-mTOR cascade in bladder cancer cells induced by MB-10.

### Intraperitoneal injection of MB-10 impedes bladder cancer xenograft growth in nude mice

**Fig. 8:**
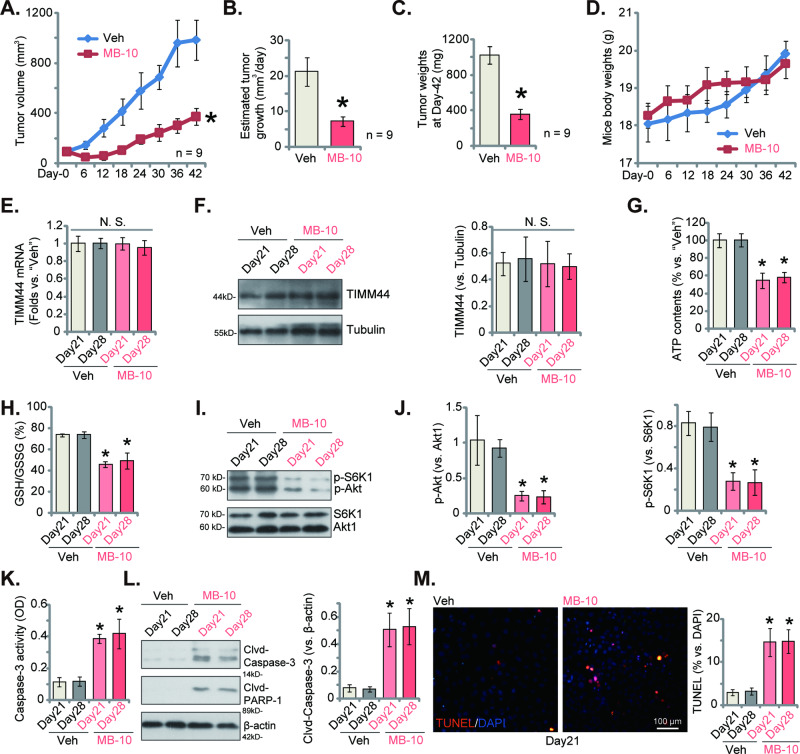
Intraperitoneal injection of MB-10 impedes bladder cancer xenograft growth in nude mice. The priBlCa-1 xenograft-bearing nude mice were intraperitoneally (*i.p*.) administrated with MB-10 (20 mg/kg body weight, given at every 48 h for a total of three doses) or vehicle control (“Veh”, PBS + Tween 80); The priBlCa-1 xenograft volumes (**A**) and animal body weights (**D**) were recorded; The estimated daily tumor growth was calculated (**B**); At Day-42, all priBlCa-1 xenografts were carefully isolated and weighed (**C**); Expression of *TIMM44* mRNA and listed proteins in the described priBlCa-1 xenograft tissues was tested (**E**, **F**, **I**, **J** and **L**). ATP contents (**G**), GSH/GSSG ratio (**H**) and Caspase-3 activity (**K**) in tissue lysates were examined as well. The priBlCa-1 xenograft slides were also subject to fluorescence detection of TUNEL-positive nuclei (**M**). The data were presented as mean ± standard deviation (SD). In (**A**–**D**) nine mice were in each group (*n* = 9). For (**E**–**M**) five random tissue pieces in each xenograft were tested (*n* = 5). *P < 0.05 vs. “Veh” group. “N. S.” stands for non-statistical difference (*P* > 0.05). Scale bar = 100 μm.

The potential anti-cancer effect of MB-10 in vivo was studied next. The priBlCa-1 primary bladder cancer cells were subcutaneously (*s.c*.) injected to the flanks of nude mice, forming xenografts after three weeks. The mice were then intraperitoneally (*i.p*.) administrated with MB-10. The first day of MB-10 injection was marked as “Day-0”. MB-10 was administrated at 20 mg/kg body weight, given at every 48 h for a total of three doses. The tumor growth curve results in Fig. 8A showed that MB-10 injection potently inhibited priBlCa-1 xenograft growth in nude mice. Xenograft volumes of MB-10-treated mice were significantly lower than those of vehicle control mice (Fig. 8A). A previously described formula [39] was employed to calculate the estimated daily priBlCa-1 xenograft growth (in mm^3^ per day) and results again showed that MB-10 impeded priBlCa-1 xenograft growth (Fig. 8B). At Day-42, all priBlCa-1 xenografts were carefully isolated from nude mice and weighed individually. MB-10-treated xenografts were significantly lighter than the vehicle-treated ones (Fig. 8C). However, there was no significant difference in animal body weights between the two groups (Fig. 8D).

At experimental Day-21 and Day-28, one priBlCa-1 xenograft was isolated from each group, and a total of four priBlCa-1 xenografts were obtained. Part of the xenograft tumors were homogenized by tissue lysis buffer. MB-10 treatment did not alter *TIMM44* mRNA (Fig. 8E)and protein (Fig. 8F) expression in priBlCa-1 xenograft tissues. ATP contents (Fig. 8G) and GSH/GSSG ratio (Fig. 8H) were however both significantly decreased in MB-10-treated priBlCa-1 xenograft tissues, supporting mitochondrial dysfunction. Moreover, Akt-S6K1 phosphorylation was largely inhibited in MB-10-treated xenografts (Fig. 8I, J). Treatment with the TIMM44 blocker also induced apoptosis activation in vivo. The caspase-3 activity (Fig. 8K), Caspase-3/PARP cleavages (Fig. 8L) and TUNEL-positive nuclei ratio (Fig. 8M) were both significantly augmented in MB-10-treated priBlCa-1 xenografts. Therefore, intraperitoneal injection of MB-10 disrupted mitochondrial function, inactivated Akt-mTOR cascade and provoked apoptosis in priBlCa-1 xenografts.

### TIMM44 KO inhibits priBlCa-1 xenograft growth in mice

**Fig. 9:**
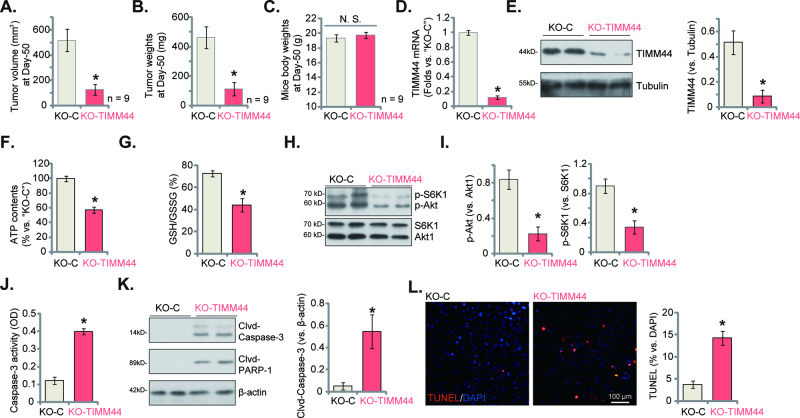
TIMM44 KO inhibits priBlCa-1 xenograft growth in mice. The priBlCa-1 primary bladder cancer cells (six million cells per mouse) with the CRISPR/Cas9-TIMM44-KO construct (“KO-TIMM44”) or the CRISPR/Cas9-KO control construct (“KO-C”) were *s.c*. injected to the nude mice. After 50 days, all priBlCa-1 xenografts of the two groups were isolated and measured (**A**, **B**). The animal body weights were recorded (**C**). Expression of TIMM44 mRNA and listed proteins in priBlCa-1 xenograft tissues was tested (**D**, **E**, **H**, **I** and **K**); ATP contents (**F**), GSH/GSSG ratio (**G**) and Caspase-3 activity (**J**) in tissue lysates were examined as well. The priBlCa-1 xenograft slides were also subjected to detection of TUNEL-positive nuclei (**L**). The data were presented as mean ± standard deviation (SD). In (**A**–**C**) nine mice were in each group (*n* = 9). For (**D**–**L**) five random tissue pieces in each xenograft were tested (*n* = 5). *P < 0.05 vs. “KO-C” group. Scale bar = 100 μm.

We further show that depletion of TIMM44 mimicked MB-10 activity in vivo. The exact same number of priBlCa-1 primary bladder cancer cells (six million cells per mouse) with the CRISPR/Cas9-TIMM44-KO construct (“KO-TIMM44”, see Fig. 5) or the CRISPR/Cas9-KO control construct (“KO-C”, see Fig. 5) were *s.c*. injected to the nude mice. Xenografts were separated 50 days after cell inoculation. As shown, KO-TIMM44 priBlCa-1 xenografts were significantly smaller (Fig. 9A) and lighter (Fig. 9B) than KO-C priBlCa-1 xenografts. Animal body weights were not significantly different among the two groups (Fig. 9C). Analyzing tumor tissues showed that *TIMM44* mRNA (Fig. 9D) and protein (Fig. 9E) levels were substantially decreased in KO-TIMM44 xenograft tissues, where ATP contents (Fig. 9F) and GSH/GSSG ratio (Fig. 9G) were decreased. Akt-mTOR activation was inhibited in KO-TIMM44 xenografts, as p-Akt and p-S6K1 levels were decreased (Fig. 9H, I). TIMM44 KO also provoked apoptosis activation in priBlCa-1 xenografts, evidenced by Caspase-3 activation (Fig. 9J), Caspase-3-PARP1 cleavage (Fig. 9K) and TUNEL-positive nuclei increase (Fig. 9L) in KO-TIMM44 xenografts.

## Discussion

To meet the energy and biosynthetic demands of tumor cell growth, mitochondrial structure and function undergo significant changes in bladder cancer, a process known as tumor mitochondrial metabolic reprogramming [14, 40]. Bladder cancer may rely more on their complete mitochondrial respiration and enhanced mitochondrial functions, which play a key role in promoting tumor cell growth and tumor progression [14, 40]. It has been shown that expression and function of a number of different mitochondria genes are altered in bladder cancer [9, 13, 41, 42].

In the present study, we show that the mitochondrial protein TIMM44 is a promising therapeutic target of bladder cancer. *TIMM44* mRNA and protein expression is significantly elevated in different human bladder cancer tissues and various bladder cancer cells. Its expression is however relatively low in normal bladder tissues and bladder epithelial cells. Genetic depletion of TIMM44, by the CRISPR-Cas9 method, produced robust anti-bladder cancer activity, inhibiting cell viability, proliferation and mobility, and provoking cell death and apoptosis. Ectopic overexpression of TIMM44 using lentiviral construct augmented bladder cancer cell growth and motility. In vivo, bladder cancer xenograft growth in nude mice was largely suppressed after genetic depletion of TIMM44.

**Fig. 10:**
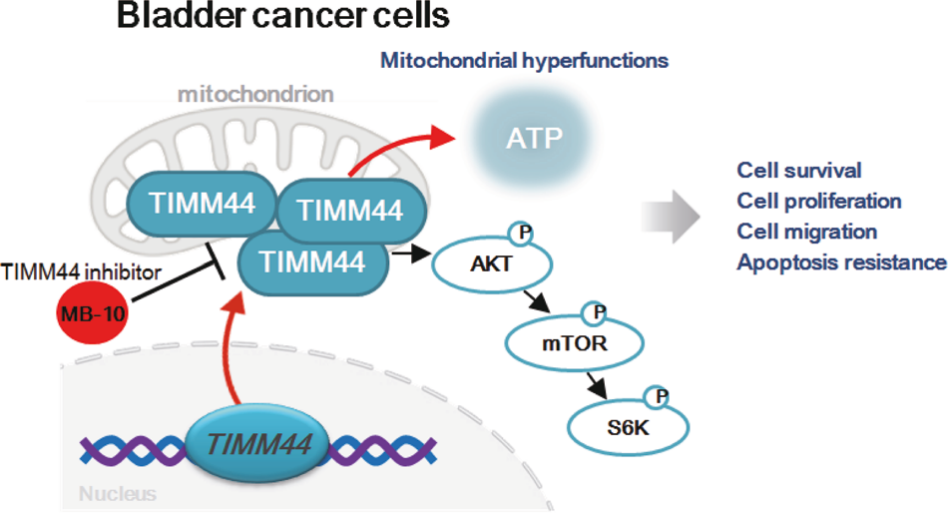
The proposed signaling carton of the present study. TIMM44 overexpression sustains heightened mitochondrial activity and promotes the Akt-mTOR pathway activation, consequently fostering the in vitro and in vivo growth of bladder cancer cells.

Importantly, the first-in-class TIMM44 blocker MB-10 [22, 23] potently inhibits bladder cancer cell growth. In both patient-derived primary bladder cancer cells and immortalized T24 cells, MB-10 exerted potent anti-cancer activity and inhibited cell viability, proliferation, migration and invasion. The TIMM44 blocker also induced apoptosis and cell cycle arrest in bladder cancer cells, yet failed to provoke cytotoxicity in primary bladder epithelial cells. In vivo, intraperitoneal injection of MB-10 impeded bladder cancer xenograft growth in nude mice (see Fig. 10).

Early studies have shown that TIMM44 is critical for maintaining mitochondrial functions and integrity. In glioma cells and endothelial cells, TIMM44 shRNA or KO resulted in mitochondrial functional impairment [21, 22], inhibiting glioma cell growth and angiogenesis [21, 22]. Here, MB-10 treatment or TIMM44 KO disrupted mitochondrial functions in bladder cancer cells, causing mitochondrial depolarization, oxidative stress and ATP reduction. Whereas exogenously-added ATP and the antioxidant NAC mitigated MB-10-induced cytotoxicity of bladder cancer cells. Oxidative stress and ATP reduction were also detected in MB-10-treated or TIMM44 KO xenograft tissues. Whereas the mitochondrial complex I activity and cellular ATP contents were both increased after TIMM44 overexpression. Therefore, TIMM44 overexpression should be important for maintaining hyper-function of mitochondria, promoting bladder cancer growth and progression (see Fig. 10).

A recent study showed that targeting TIMM44 by a mitochondria-targeting near-infrared (NIR) fluorophore IR-58 activated autophagy and apoptosis in an mTOR-related manner, thereby inhibiting colorectal cancer growth [43]. Here we found that MB-10 also inhibited mTOR and activated autophagy in priBlCa-1 cells, yet autophagy inhibitors failed to mitigate MB-10-induced cytotoxicity in bladder cancer cells. Therefore the mechanism of action of MB-10-induced cytotoxicity in bladder cancer cells may be different from that of IR-58 targeting TIMM44 in colorectal cancer cells. The ineffectiveness of autophagy inhibitors in mitigating cytotoxicity of MB-10 could be due to the marginal role of autophagy in cell survival/apoptosis and/or the existence of compensatory mechanisms within these cells.

Recent studies have shown that disruption of mitochondrial functions can lead to Akt-mTOR inactivation in different cancer cells. The mitochondrial protein aarF domain containing kinase 2 (ADCK2) is essential for fatty acid metabolism and coenzyme Q biosynthesis [44]. Zhang et al., reported that ADCK2 shRNA or KO (via CRISPR/Cas9 method) inhibited Akt-mTOR activation in non-small cell lung cancer cells [45]. Contrarily, ADCK2 overexpression enhanced mitochondrial functions and Akt-mTOR cascade activation [45]. Han et al., showed that genetic depletion of TIMM13 (translocase of inner mitochondrial membrane 13) provoked mitochondrial dysfunction and inhibited Akt-mTOR activation in osteosarcoma cells [46]. Xia et al., also demonstrated that overexpression of the mitochondrial protein YME1 Like 1 (YME1L) promoted Akt-mTOR cascade activation in NSCLC cells [47] and YME1L silencing or KO decreased signaling [47]. Interestingly, cancer cells exhibiting hyperactive Akt showcased elevated levels of ROS. Marchi et al., reported that Akt-induced phosphorylation of the mitochondrial calcium uniporter (MCU) regulatory subunit led to increased basal mitochondrial Ca^2+^ levels, causing ROS production and tumor progression [48]. Akt activation was shown to induce premature senescence and sensitize cells to ROS-mediated apoptosis by increasing intracellular ROS contents [49]. PTEN-deficient prostate cancer cells with hyperactive Akt exhibited elevated ROS levels, partially due to an Akt-dependent enhancement of oxidative phosphorylation [50].

In the present study, we showed that TIMM44 is important for Akt-mTOR activation in bladder cancer cells. In priBlCa-1 primary bladder cancer cells, Akt-S6K1 phosphorylation was largely decreased by MB-10 treatment or TIMM44 KO. It was however enhanced after ectopic TIMM44 overexpression. Akt-mTOR activation was also largely inhibited in MB-10-treated priBlCa-1 xenograft tissues or TIMM44 KO priBlCa-1 xenograft tissues. Importantly, restoring Akt-mTOR activation by caAkt1 mitigated MB-10-induced cytotoxicity in priBlCa-1 cells. In our study, the addition of exogenous ATP alleviated MB-10-induced inactivation of Akt-mTOR cascade in priBlCa-1 cells, suggesting that reduction in ATP levels could be the primary mechanism underlying the inhibition of the Akt-mTOR cascade by MB-10 in bladder cancer cells (see Fig. 10). These results support that mediating Akt-mTOR cascade activation is one important mechanism of TIMM44-driven bladder cancer growth (see Fig. 10).

### Supplementary information

Supplemental Figures S1-S2

Original Data File

aj-checklist form

## Data Availability

All data are available upon request.
