# Supplementary material for: A first-in-class TIMM44 blocker inhibits bladder cancer cell growth
Source: Cell Death Dis. 2024 Mar 11;15(3):204. doi: 10.1038/s41419-024-06585-x (PMC10928220; doi:10.1038/s41419-024-06585-x)
Supplement: Supplementary file 1 — Supplemental Figures S1-S2 [file 41419_2024_6585_MOESM1_ESM.pdf]

Figure S1. The uncropped blotting images

Figure 1.

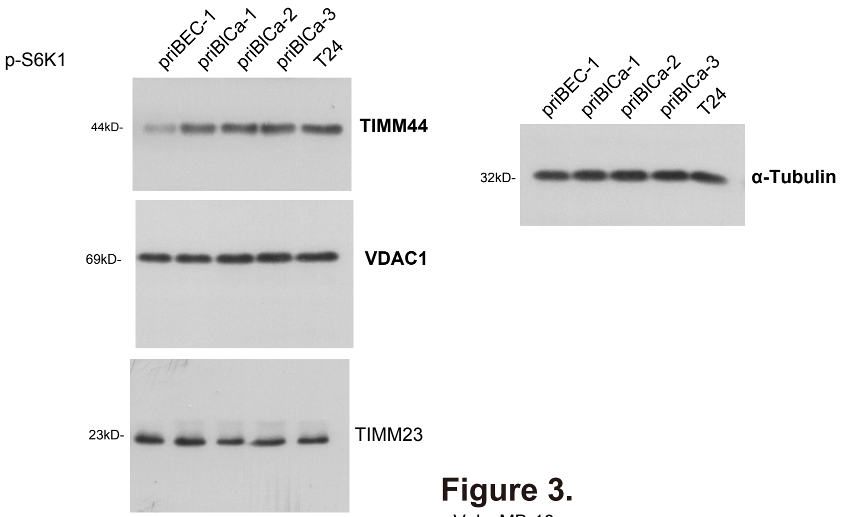

Figure 3.

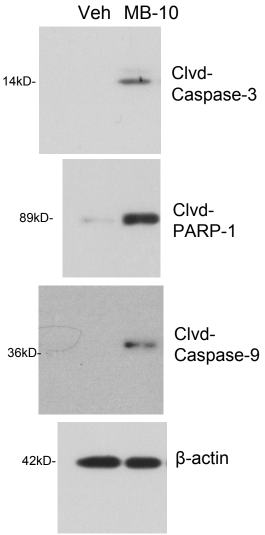

Figure 2.

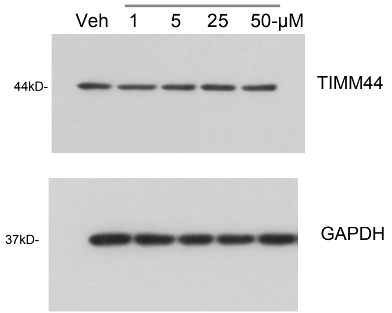

Figure 5.

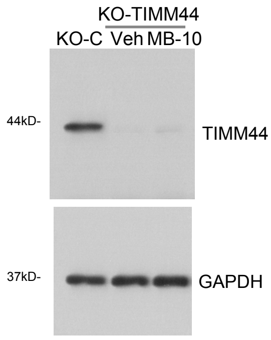

Figure 6.

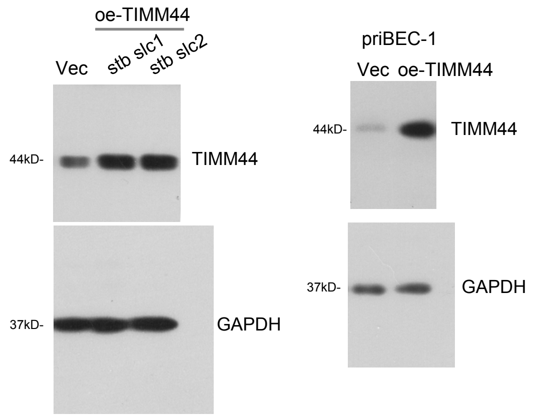

Figure 8.

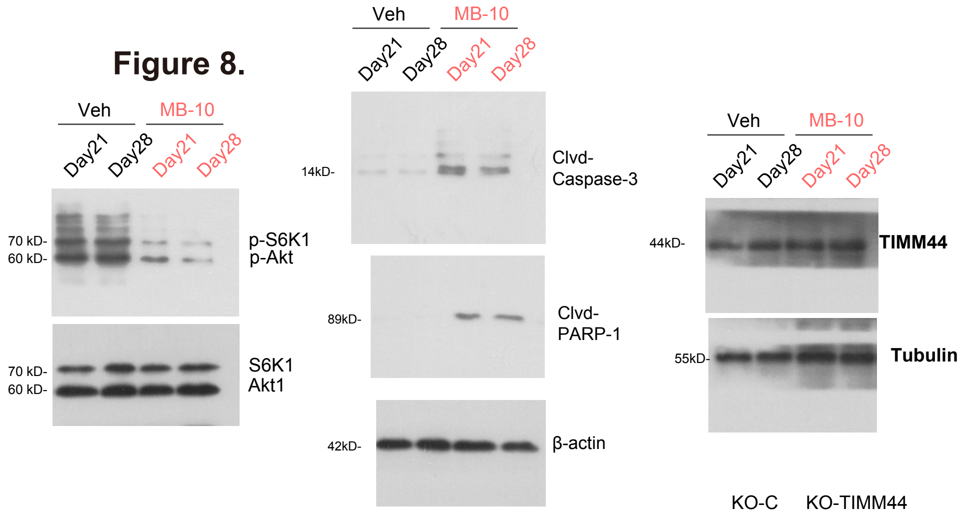

Figure 9.

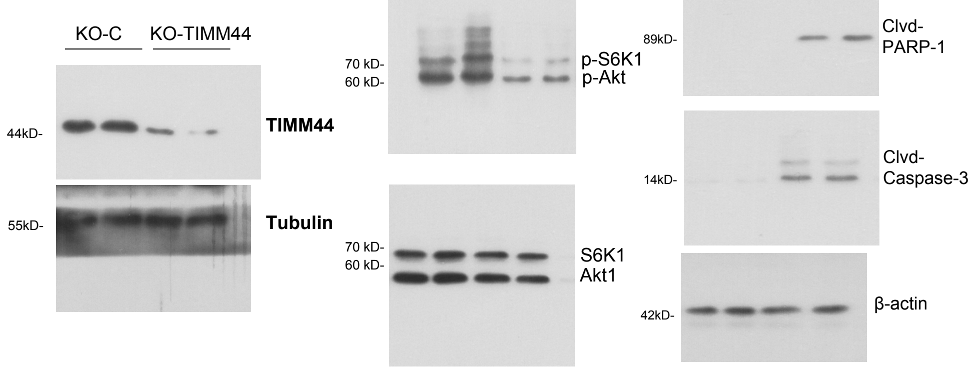

Figure 7.

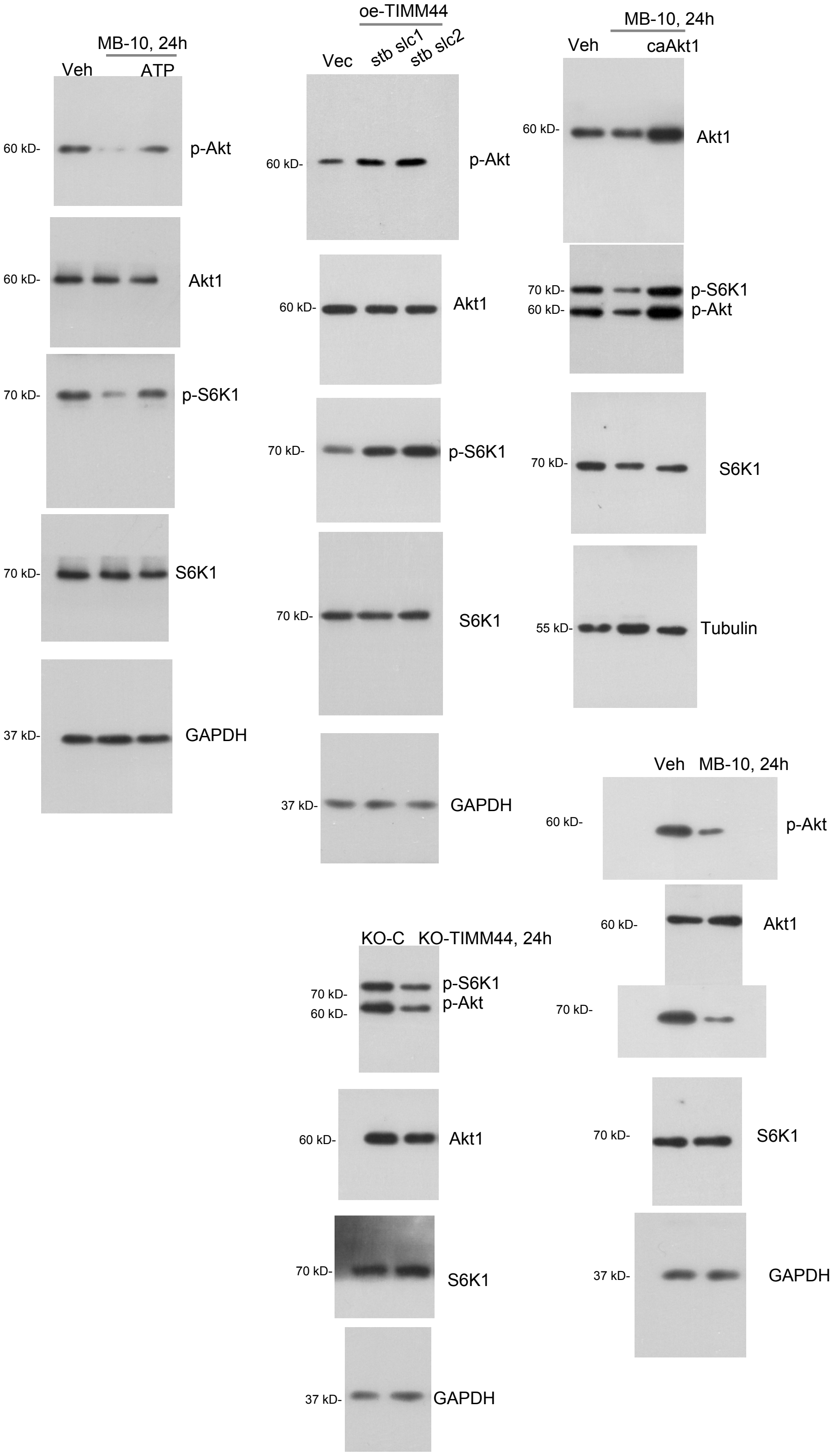
MB-10, 24h
Veh
ATP
60 kD-
p-Akt
60 kD-
Akt1
70 kD-
p-S6K1
70 kD-
S6K1
37 kD-
GAPDH
oe-TIMM44
Vec
stb slc1
stb slc2
60 kD-
p-Akt
60 kD-
Akt1
70 kD-
p-S6K1
70 kD-
S6K1
37 kD-
GAPDH
MB-10, 24h
Veh
caAkt1
60 kD-
Akt1
70 kD-
60 kD-
p-S6K1
p-Akt
70 kD-
S6K1
55 kD-
Tubulin
Veh
MB-10, 24h
60 kD-
p-Akt
60 kD-
Akt1
70 kD-
70 kD-
S6K1
37 kD-
GAPDH
KO-C
KO-TIMM44, 24h
70 kD-
60 kD-
p-S6K1
p-Akt
60 kD-
Akt1
70 kD-
S6K1
37 kD-
GAPDH

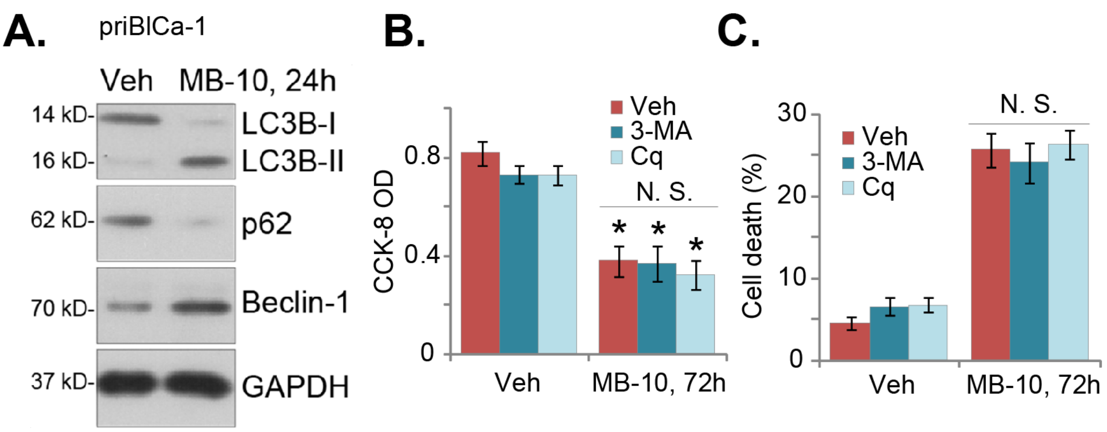

Figure S2. The priBlCa-1 primary bladder cancer cells were maintained under complete medium, treated with MB-10 (25 μ M), and cultivated for 24h, expression of listed proteins was shown (**A**, representing five different repeats). The priBlCa-1 cells were pretreated for 1h with 3-methyladenine (3-MA, 5 mM) or chloroquine (Cq) (25 μ M), followed by MB-10 (25 μ M) stimulation, cells were further cultivated for indicated time periods, cell viability and death were tested by CCK-8 (**B**) and Trypan blue staining (**C**) assays, respectively. "Veh" stands for the vehicle control treatment (0.1% DMSO). The data were presented as mean ± standard deviation (SD). * ***P*** < 0.05 *vs.* "Veh". "N.S." stands for non-statistical difference (***P*** > 0.05). The *in vitro* experiments were repeated five times with similar results obtained.
