# Supplementary material for: A first-in-class TIMM44 blocker inhibits bladder cancer cell growth
Source: Cell Death Dis. 2024 Mar 11;15(3):204. doi: 10.1038/s41419-024-06585-x (PMC10928220; doi:10.1038/s41419-024-06585-x)
Supplement: Supplementary file 2 — Original Data File [file 41419_2024_6585_MOESM2_ESM.pdf]

Figure S1. The uncropped blotting images

Figure 1.

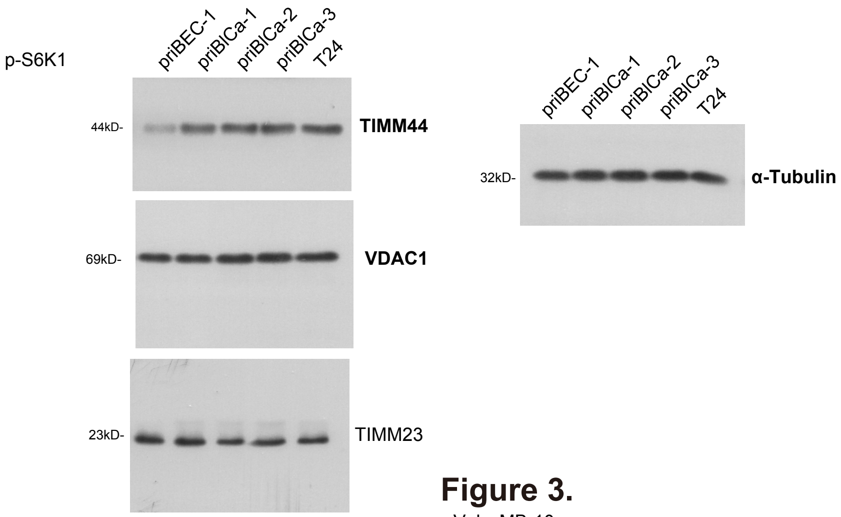

Figure 2.

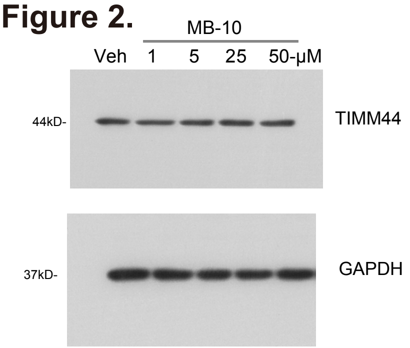

Figure 3.

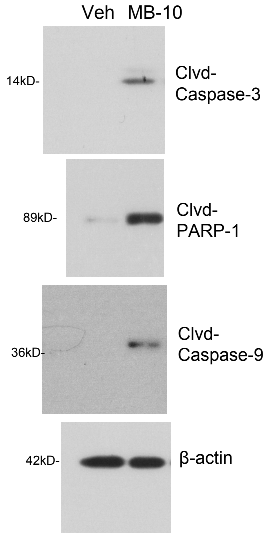

Figure 5.

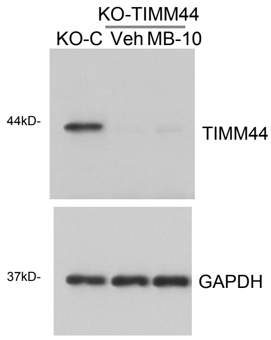

Figure 6.

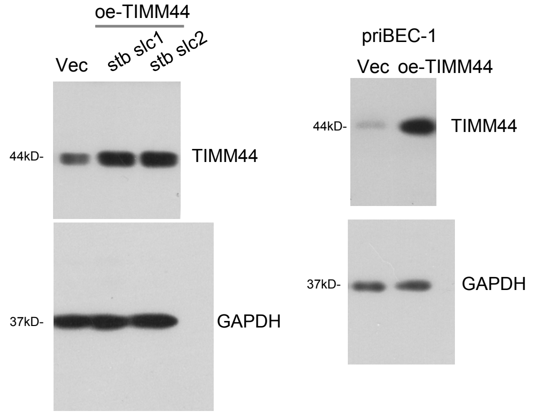

Figure 8.

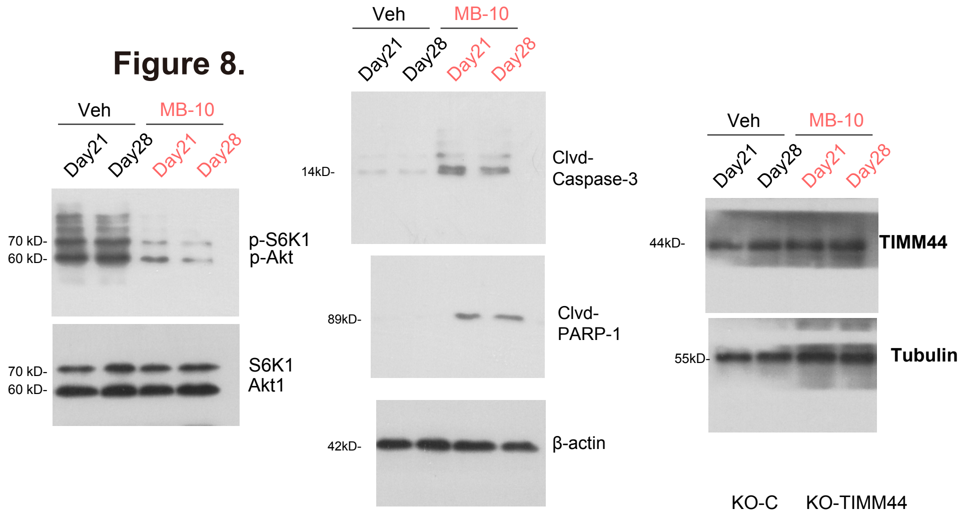

Figure 9.

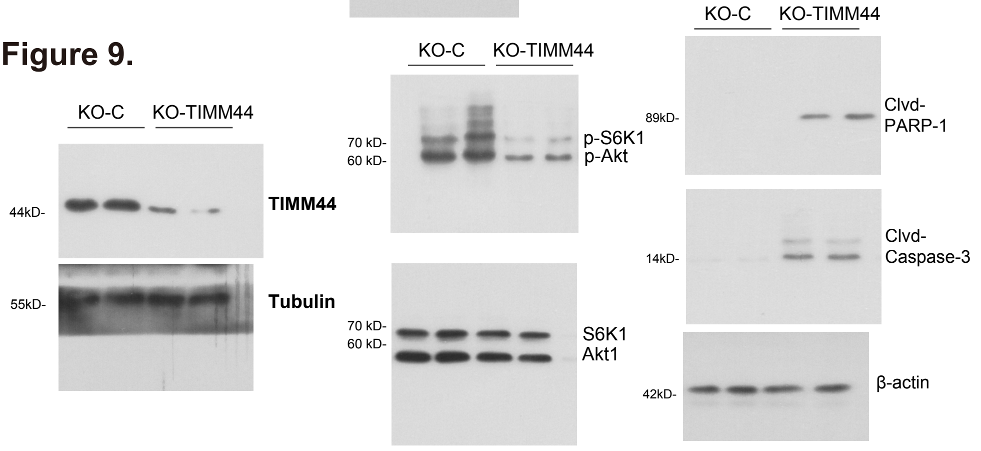

Figure 7.

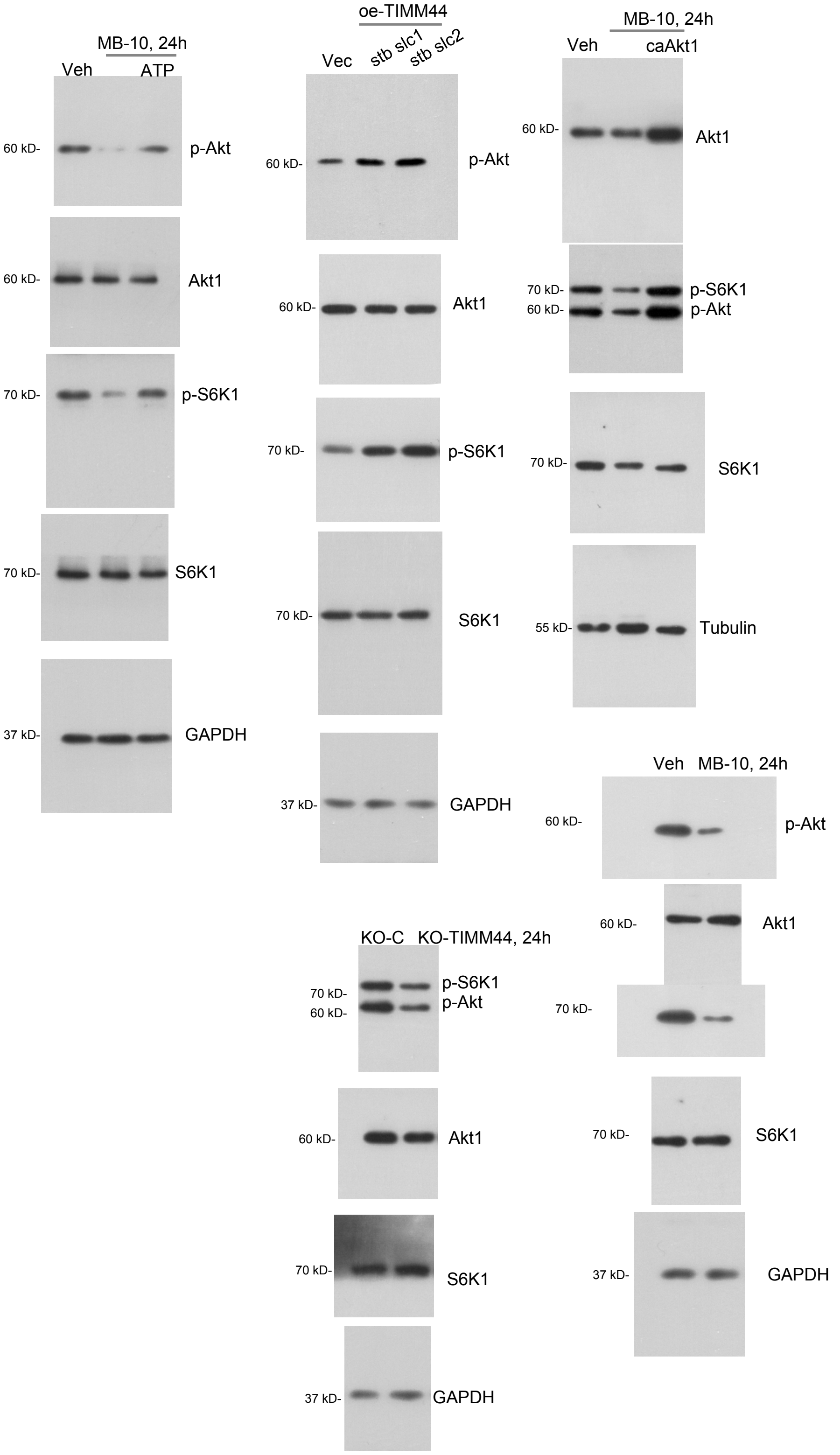
MB-10, 24h
Veh
ATP
60 kD-
p-Akt
60 kD-
Akt1
70 kD-
p-S6K1
70 kD-
S6K1
37 kD-
GAPDH
oe-TIMM44
Vec
stb slc1
stb slc2
60 kD-
p-Akt
60 kD-
Akt1
70 kD-
p-S6K1
70 kD-
S6K1
37 kD-
GAPDH
MB-10, 24h
Veh
caAkt1
60 kD-
Akt1
70 kD-
60 kD-
p-S6K1
p-Akt
70 kD-
S6K1
55 kD-
Tubulin
Veh
MB-10, 24h
60 kD-
p-Akt
60 kD-
Akt1
70 kD-
70 kD-
S6K1
37 kD-
GAPDH
KO-C
KO-TIMM44, 24h
70 kD-
60 kD-
p-S6K1
p-Akt
60 kD-
Akt1
70 kD-
S6K1
37 kD-
GAPDH
